# Homoharringtonine suppresses imatinib resistance via the Bcl-6/p53 pathway in chronic myeloid leukemia cell lines

**DOI:** 10.18632/oncotarget.16731

**Published:** 2017-03-30

**Authors:** Qian Wang, Wei Ding, Yihan Ding, Jingjing Ma, Zhaoye Qian, Jingxian Shao, Yufeng Li

**Affiliations:** ^1^ Department of Hematology, Huai’an First People's Hospital, Nanjing Medical University, Huai’an, 223300, China; ^2^ Department of Oncology, Huai’an First People's Hospital, Nanjing Medical University, Huai’an, 223300, China

**Keywords:** Bcl-6/p53 pathway, homoharringtonine, imatinib, drug-resistance, chronic myeloid leukemia

## Abstract

**Background:**

The anti-leukemic mechanism of homoharringtonine (HHT) differs from that of IM, and HHT is one of the most useful agents for use in patients with IM resistance or intolerance. The Bcl-6/p53 pathway has been shown to regulate the sensitivity of tumor cells to antitumor drugs. We tested whether HHT blocked the Bcl-6/p53 pathway in order to promote the apoptosis of IM-resistant cells *in vitro* and *in vivo*.

**Results:**

Ph+ acute lymphoblastic leukemia (ALL) cells and IM-resistant chronic myeloid leukemia (CML) cells showed high expression of Bcl-6 protein. Bcl-6 mediated the upregulation of p53, and and Bcl-6 induced growth inhibition of IM-resistant cells as well as its apoptosis by targeting p53. In addition, Bcl-6 was downregulated moderately after HHT treatment in different cells. The Bcl-6 expression was significantly increased in patients with CML when compared with healthy subjects. Furthermore, the expression of Bcl-6 was higher in patients with CML-blastic phase (CML-BP) than in those with CML-chronic phase (CML-CP).

**Methods:**

The inhibitory effect of drugs on cell growth was detected by Cell Counting Kit-8 (CCK-8), The apoptosis rate and the cell cycle were investigated by flow cytometry. The expression of Bcl-6, p53, Bcl-2, caspase9, and caspase3 proteins was assayed by western blot, Real- Time PCR (qPCR) detect *Bcl-6* and *p53* mRNA.

**Conclusions:**

HHT can suppress the growth and induce apoptosis of IM-resistant cells, the mechanism of which is associated with blocking of the Bcl-6/p53 pathway. Our results could offer a theoretical explanation for HHT use in patients with IM resistance or intolerance.

## INTRODUCTION

Chronic myeloid leukemia (CML) arises from pluripotent hemopoietic stem cells with a reciprocal translocation between chromosomes 9 and 22 [1]. The chromosomal abnormality persistently activates tyrosine kinase, resulting in the activation of a downstream signaling pathway that inhibits cell apoptosis [2–4]. In 2002, the US Food and Drug Administration (FDA) approved the tyrosine kinase inhibitor (TKI) imatinib (IM) for CML treatment [5]. The international randomized study (IRIS) reported a complete hematologic remission rate of 97% [6], complete cytogenetic response rate of 63% [7], and major cytogenetic response rate of 87% [8] in newly diagnosed CML-chronic phase (CML-CP) patients after 12 months of IM treatment. Only 7% of patients entered accelerated phase (AP) disease or blast crisis (BC) [9]. Although IM is highly effective for treating CML, it does have limitations. These include an inability to eradicate leukemia stem cells (LICs) and the occurrence of primary or secondary drug resistance [10–12]. IRIS reported an overall incidence of IM resistance of 18% at 5 years [13]. Compared with CML-CP patients, patients with progressive disease had higher drug resistance and relapse rates. The drug resistance rate was 75% CML-AP and 95% in CML-BP patients [14]. Recent studies have reported similar findings [15].

The B-cell lymphoma (*Bcl-6*) gene encodes a 96 kD nucleophosmin (NPM) that belongs to the bric-a-brac tramtrack broad complex/pox virus zinc (BTB/POZ) transcription factor family [16]. The functional Bcl-6 protein is a transcription repressor containing 706 amino acids, and the inhibition of *Bcl-6* transcription results from the interaction of BTB/POZ with a number of corepressors [17, 18]. *Bcl-6* is closely associated with the occurrence and development of diffuse large B-cell lymphoma (DLBCL) and follicular lymphoma (FL) [19]; 20%–40% of DLBCL and 15% of FL patients have the *Bcl-6* 3q27 chromosome translocation [20, 21]. Bcl-6 can suppress p53 expression in germinal center B-cell like DLBCL and inhibit B-cell apoptosis caused by DNA damage. Ryan et al. found that Bcl-6 could downregulate p53 by binding to its promoter region [22].

The anti-leukemic mechanism of homoharringtonine (HHT) differs from that of IM, and HHT is one of the most useful agents for use in patients with IM resistance or intolerance [23]. HHT is an inhibitor for protein translation, which blocks the synthesis of protein via affecting the A site in ribosome [24]. In October 2012, the US FDA approved the use of HHT for the treatment of CML, which gave the drug widespread attention [25].

This present study investigated the effect of HHT on the proliferation, apoptosis and cell cycle of IM-resistant CML cells and involvement of the Bcl-6/p53 signaling pathway.

## RESULTS

### The drug resistance of K562/G01 cells

**Figure 1:**
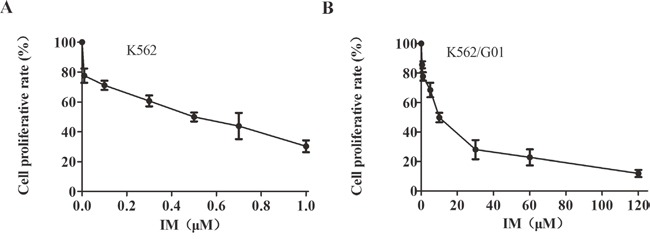
Cell growth inhibition and cytotoxicity of IM in K562 cells and K562/G01 cells **(A)** Cell growth inhibition and viability of K562 cells. K562 cells were treated with IM at the indicated concentrations for 24 hours. **(B)** Cell growth inhibition and viability of K562/G01 cells. K562/G01 cells were treated with IM at the indicated concentrations for 24 hours. Cell viability was determined by CCK-8. Values shown are mean ± SD. Of three independent experiments.

Various concentrations of IM treated K562 cells and K562/G01 cells for 24h. The K562 cells were more sensitive to IM than the K562/G01 cells. Treatment with 0.5 μM IM for 24 h induced more than 50% of K562 cells to the death (Figure 1A). Treatment with 9.5 μM IM for 24 h induced more than 50% of K562/G01cells to the death (Figure 1B). Our results show that the drug resistance of K562/G01 cells is 19 times to the K562 cells, which proves that our drug resistance cells are effective.

### Bcl-6 regulates p53 in K562/G01 cells

**Figure 2:**
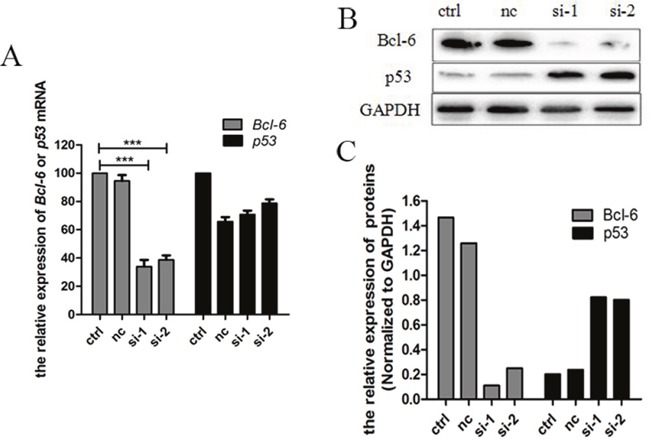
Bcl-6 mediated the upregulation of p53 in K562/G01 cells **(A)** K562/G01 cells treated with *Bcl-6* siRNA1 and siRNA2 for 48h. The levels of *Bcl-6* mRNA were (30.67±0.82)% and (38.74 ±1.76)% respectively, compared with control. The *p53* mRNA was slightly downregulated. **(B)** K562/G01 cells treated with *Bcl-6* siRNA1 and siRNA2 for 48h. The Bcl-6 protein reduced obviously. And si-1 and si-2 show that p53 protein was upregulated distinctly. **(C)** The relative expression of Bcl-6 and p53 proteins. The expression of mRNA was determined by qPCR. The expression of proteins were determined by western blot. Values shown are mean ± SD. Of three independent experiments.

In order to observe the influence of Bcl-6 on p53, we examined Bcl-6 and p53 following treatment of K562/G01 cells with *Bcl-6* siRNA. In cells treated with *Bcl-6* siRNA1 and siRNA2 for 48 h, the level of *Bcl-6* mRNA was (30.67±0.82)% and (38.74 ±1.76)%, respectively (*P* < 0.01; Figure 2A). Furthermore, after *Bcl-6* siRNA treatment, the Bcl-6 protein was obviously reduced (*P* <0.01; Figure 2B, 2C), which reveals that the downregulation of Bcl-6 was effective. Subsequently, *p53* mRNA and protein were detected. The results showed that p53 protein was upregulated distinctly (Figure 2B, 2C), while the mRNA was slightly downregulated (Figure 2A). Therefore, Bcl-6 mediated the upregulation of p53 in K562/G01 cells.

### K562/G01 cells are sensitive to Bcl-6-induced growth inhibition and apoptosis

**Figure 3:**
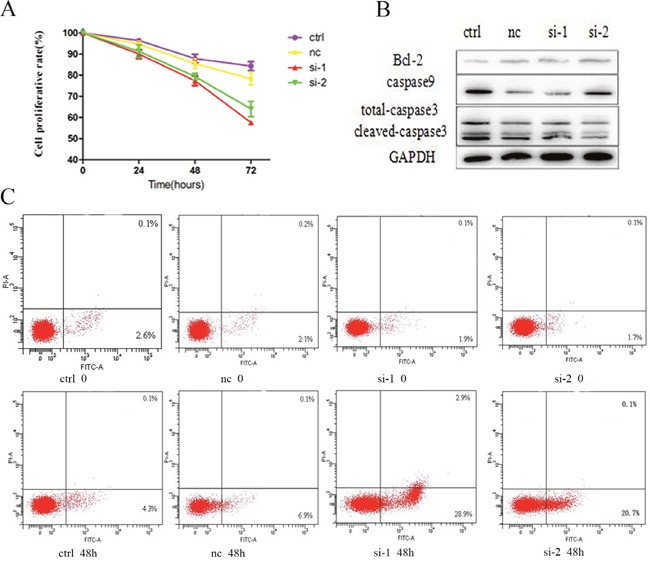
K562 /G01 cells are sensitive to Bcl-6-induced growth inhibition and apoptosis **(A)** Cell growth inhibition and viability of K562/G01 cells. K562/G01 cells treated with *Bcl-6* siRNA1 and siRNA2 for 24h, 48h, 72h. **(B)** The expression of apoptosis proteins. K562/G01 cells treated with *Bcl-6* siRNA1 and siRNA2 for 48h. Bcl-2, caspase9, caspase3 express. **(C)** Apoptosis of K562/G01 cells. K562/G01 cells treated with *Bcl-6* siRNA1 and siRNA2 for 48h. Cell viability was determined by CCK-8. The expression of proteins were determined by western blot. PE annexin V detect apoptosis. Values shown are mean ± SD. Of three independent experiments.

After downregulation of Bcl-6, we investigated the cell growth and apoptosis of K562/G01 cells at 24, 48, and 72 h. The results showed that downregulation of Bcl-6 can inhibit K562/G01 cell growth, in a time-dependent mode (Figure 3A). The data are shown in Table 1. We also assessed the effect of Bcl-6 on cell apoptosis. After 48 h, the apoptosis rate was (4.50±0.17)%, (7.23±0.25)%, (30.9±1.67)%, and (23.26±1.61)%, respectively (Figure 3C, Table 2). Furthermore, we detected the apoptosis-related proteins. Bcl-2 and total caspase9 were reduced, and cleaved-caspase3 was upregulated (Figure 3B). In summary, we suggest that K562/G01 cells are sensitive to Bcl-6-induced growth inhibition and apoptosis.

**Table 1:** Cell proliferative rate of K562/G01 cells treated with *Bcl-6* siRNA1 and siRNA2 respectively (n=3, mean ± SD)

Cell proliferative rate (%)				
Time(h)	ctrl	nc	si-1	si-2
0	100.00	100.00	100.00	100.00
24	96.46±0.45	94.70±0.87	90.03±1.17	91.35±1.76
48	87.86±1.16	85.53±1.34	77.39±1.31**	79.41±0.92**
72	84.33±1.20	78.33±1.66	57.57±0.03***	64.04±2.04**

**P<0.01,***P<0.001, compared with ctrl.

**Table 2:** Apoptosis rate of K562/G01 cells treated with *Bcl-6* siRNA1 and siRNA2 respectively (n=3, mean ± SD)

Apoptosis rate (%)				
Time(h)	ctrl	nc	si-1	si-2
0	2.80±0.10	2.46±0.15	2.10±0.26	1.96±0.20
48	4.50±0.17	7.23±0.25	30.90±1.67***	23.26±1.61**

**P<0.01,***P<0.001, compared with ctrl.

### HHT-induced K562/G01 cells growth inhibition, apoptosis and cell cycle arrest

**Figure 4:**
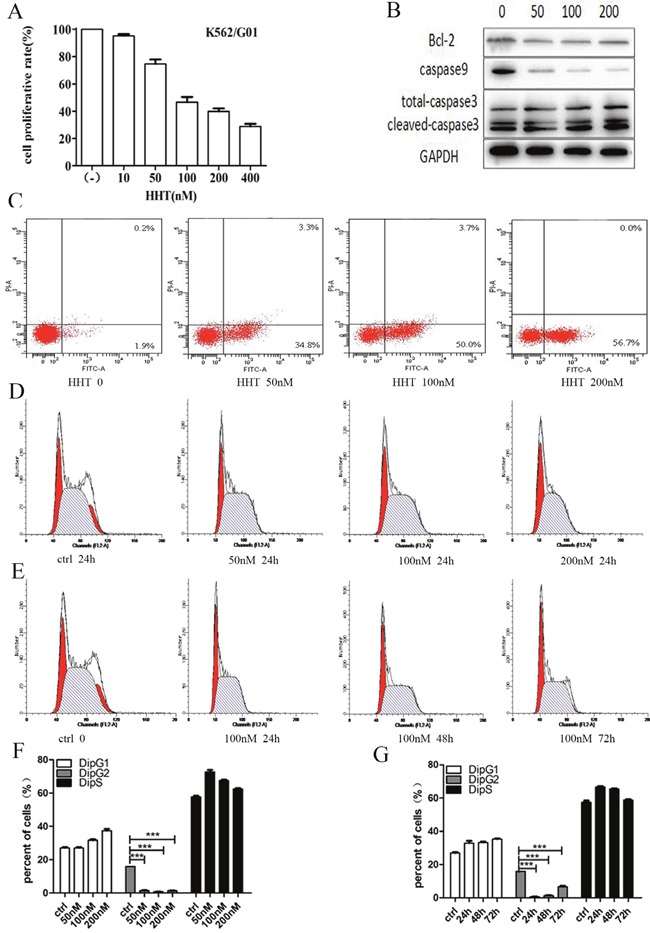
Cell growth inhibition, apoptosis and cell cycle arrest of HHT in K562/G01 cells **(A)** K562/G01 cells were treated with HHT at the indicated concentrations for 24 hours. The concentrations were 0, 10, 50, 100, 200, 400 nM. **(B)** The expression of apoptosis proteins. K562/G01 cells treated with HHT 0, 50nM, 100 nM, 200nM for 24h. Bcl-2, caspase9, caspase3 express. **(C)** Apoptosis of K562/G01 cells. K562/G01 cells treated with HHT 0, 50nM, 100 nM, 200nM for 24h. **(D)** Cycle of K562/G01 cells. K562/G01 cells treated with HHT 0, 50nM, 100 nM, 200nM for 24h. **(E)** Cycle of K562/G01 cells. K562/G01 cells treated with 100 nM HHT for 24h, 48h, 72h. **(F)** Cycle of K562/G01 cells. K562/G01 cells treated with HHT 0, 50nM, 100 nM, 200nM for 24h. **(G)** Cycle of K562/G01 cells. K562/G01 cells treated with 100 nM HHT for 24h, 48h, 72h. Cell viability was determined by CCK-8. The expression of proteins were determined by western blot. PE annexin V detect apoptosis. Flow cytometry detect cycle. Values shown are mean ± SD. Of three independent experiments.

The cells were treated with 0, 10, 50, 100, 200, or 400 nM HHT for 24 h. The results showed that HHT can inhibit K562/G01 cell growth, in a time-dependent mode (Figure 4A). It suggests that HHT could induce K562/G01 cells growth inhibition. We also assessed the effects of HHT on cell apoptosis. In K562/G01 cells cultured with 0, 50, 100, or 200 nM HHT for 24 h, respectively, the rate of apoptosis was (1.93 ±0.15)%, (35.50 ±1.47)%, (48.80±1.10)%, and (53.50±0.62)%, respectively (Figure 4C, Table 3). Furthermore, we investigated the apoptosis-related proteins: Bcl-2 and total caspase9 were reduced, and cleaved-caspase3 was upregulated (Figure 4B). We observed that HHT could induce apoptosis in IM-resistant cells, and that HHT inhibited the cell cycle progression of K562/G01 cells. The cell cycle distribution was analyzed by flow cytometry after treatment with HHT at different doses and for different time. The cells were treated with 0, 50, 100, or 200 nM HHT for 24 h. The results suggested that HHT blocks the G1 phase change from the G2 period. The cell number was distinctly decreased after HHT treatment, when compared with control (*P* < 0.001; Figure 4D, 4F). The cells were treated with 100 nM HHT for 24, 48, or 72 h. The results suggested that HHT blocks the G1 phase change from the G2 period, as shown previously. The cell number was distinctly decreased after an increased time, when compared with control (*P* < 0.001; Figure 4E, 4G).

**Table 3:** Apoptosis rate of K562/G01 cells treated with HHT 0, 50nM, 100nM, 200nM respectively (n=3, mean± SD)

Apoptosis rate (%)				
Time(h)	0	50nM	100nM	200nM
24	1.93 ± 0.15	35.50 ± 1.47**	48.80 ± 1.10**	53.50 ± 0.62***

**P<0.01,***P<0.001, compared with ctrl.

### Expression of Bcl-6 in bone marrow

**Figure 5:**
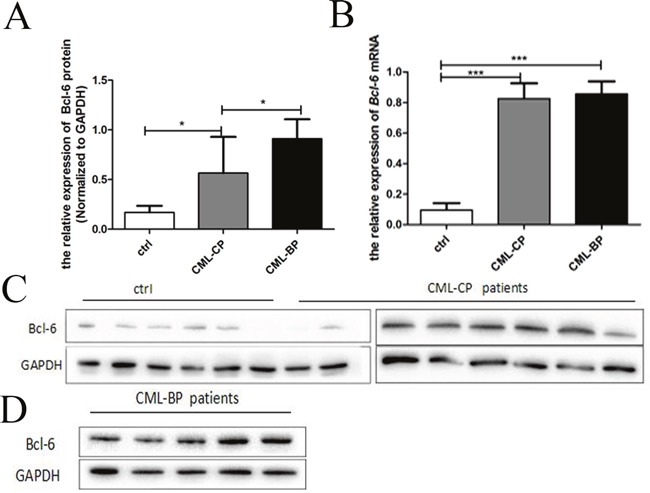
The expression of Bcl-6 in CML-CP and CML-BP patients **(A)** qRT-PCR analysis of the relative expression of *Bcl-6* mRNA in CML-CP patients and CML-BP patients and controls. **(B)** Western blot analysis of Bcl-6 protein in CML-CP patients and CML-BP patients and controls. **(C)** Western blot analysis of Bcl-6 protein in CML-CP patients and controls. **(D)** Western blot analysis of Bcl-6 protein in CML-BP patients. Values shown are mean ± SD.

Twenty CML-CP patients and ten CML-BP patients were the volunteers for samples. Table 4 is the specific information for patients. The average value of *Bcl-6* mRNA in healthy controls, CML-CP and CML-BP patients was (0.10±0.02), (0.83±0.03), (0.86 ±0.04), respectively (Figure 5B). Whereas the average value of Bcl-6 protein in healthy controls, CML-CP and CML-BP patients was (0.17±0.02), (0.57±0.12), (0.91±0.08), respectively (Figure 5A). The western blot findings are shown in Figure 5C and 5D. Above data indicates that the level of Bcl-6 protein is higher at CML-BP patients than CML-CP patients.

**Table 4:** Patient characteristics

Characteristic		CML-CP patients (n=20)	CML-BP patients (n=10)
Gender	Male	7	5
	Female	13	5
Age (years)	Median	44	53.5
	Range	23-64	21-64
WBC,×109/L	Median	42.16	180.64
	Range	2-299.88	10-434.6
Hemoglobin, g/L	Median	108	101
	Range	69-147	79-129
Platelet count, ×109/L	Median	300.5	829
	Range	41-1443	181-1238

### Bcl-6 protein in Ph+ cells, and HHT blockage of the Bcl-6/p53 pathway

**Figure 6:**
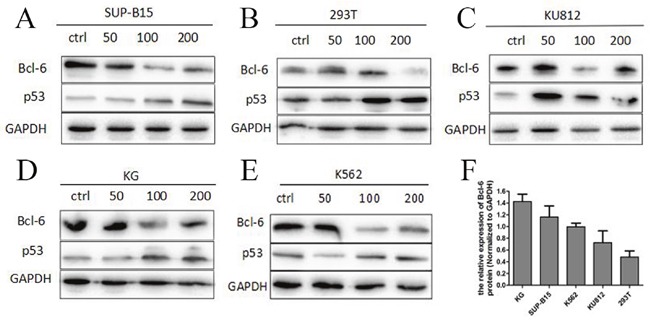
High expression of Bcl-6 protein in Ph+ cells and HHT blocked the Bcl-6/p53 pathway **(A)** SUP-B15 cells were treated with HHT 0, 50nM, 100 nM, 200nM for 24h. **(B)** 293T cells were treated with HHT 0, 50nM, 100 nM, 200nM for 24h. **(C)** KU812 cells were treated with HHT 0, 50nM, 100 nM, 200nM for 24h. **(D)** K562/G01 cells were treated with HHT 0, 50nM, 100 nM, 200nM for 24h. **(E)** K562 cells were treated with HHT 0, 50nM, 100 nM, 200nM for 24h. **(F)** The Bcl-6 expression of 5 cells without treatment. The expression of proteins were determined by Western blot. Values shown are mean ± SD. Of three independent experiments.

We tested Bcl-6 protein in K562, K562/G01, KU812, SUP-B15, and 293T cells after incubation with HHT at 0, 50 or 100 nM for 24 h. Western blot detect the expressions of Bcl-6 and p53 proteins. The results showed that the level of Bcl-6 protein declined in K562/G01 cells, K562cells, KU812 cells, 293T cells, and SUP-B15 cells, accompanied by a distinct rise in p53 (Figure 6A–6E). These results strongly suggest that HHT specifically reduces Bcl-6 in cells, and its downstream target p53. To confirm the baseline value of Bcl-6 in CML cells, we selected five cell lines in which to detect Bcl-6 protein. The level of Bcl-6 protein, from high to low, was found to vary in K562/G01 cells, SUP-B15 cells, K562 cells, KU812 cells, and 293T cells (Figure 6F).

### Effect of HHT on *Bcl-6* and *p53* mRNA expression

**Figure 7:**
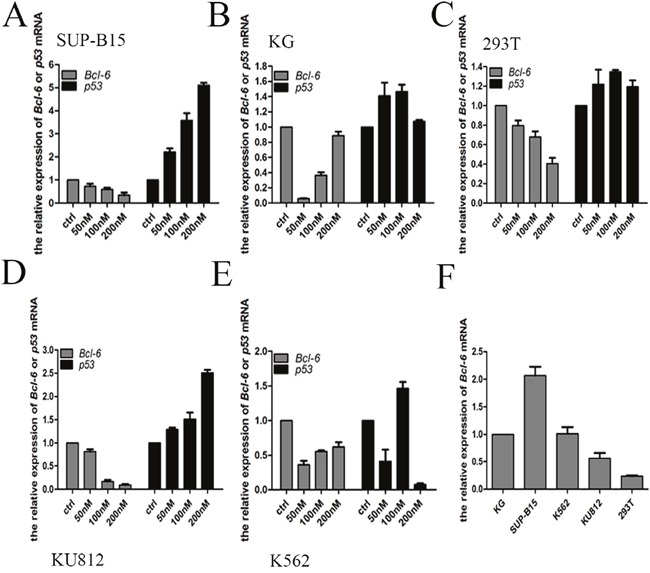
HHT effect the Bcl-6 and p53 mRNA expression **(A)** SUP-B15 cells were treated with HHT 0, 50nM, 100 nM, 200nM for 24h. **(B)** K562/G01 cells were treated with HHT 0, 50nM, 100 nM, 200nM for 24h. **(C)** 293T cells were treated with HHT 0, 50nM, 100 nM, 200nM for 24h. **(D)** KU812 cells were treated with HHT 0, 50nM, 100 nM, 200nM for 24h. **(E)** K562 cells were treated with HHT 0, 50nM, 100 nM, 200nM for 24h. **(F)** The *Bcl-6* expression of 5 cells without treatment. The expression of mRNA were determined by qRT-PCR. Values shown are mean ± SD. Of three independent experiments.

Real-Time PCR (qPCR) detect the levels of mRNA. With regard to the expression of *Bcl-6* and *p53* mRNA, the results differed from those for the expression of proteins. In KU812 and SUP-B15 cells, the level of *Bcl-6* mRNA was reduced in a dose-dependent mode after treating with HHT, accompanied by a distinct rise in *p53* (Figure 7A, 7D). In 293T cells, the amount of *Bcl-6* mRNA reduced in a dose-dependent mode after treating with HHT, whereas *p53* was upregulated irregularly (Figure 7C). In K562/G01 cells, the *Bcl-6* was downregulated compared with control, but there was a dose-dependent rise in *Bcl-6* mRNA, as in K562 cells (Figure 7B, 7E). The level of *p53* changed irregularly in K562/G01 cells and K562 cells (Figure 7B, 7E). To confirm the baseline value of *Bcl-6* in CML cells, we selected five cell lines for detection expression of *Bcl-6* mRNA. The level of expression of *Bcl-6* mRNA, from high to low, was found to vary in SUP-B15 cells, K562/G01 cells, K562 cells, KU812 cells, and 293T cells (Figure 7F).

## DISCUSSION

In 2011, Pellicano F et al described the pharmacological inhibition of Bcl-6 as a new strategy to eradicate LICs in CML [26]. Christian et al. reported that CML cells upregulated Bcl-6 in response to treatment with a TKI. They also confirmed that Bcl-6 directly repressed p53 in human CML cells [27]. Duy et al. reported that the growth of TKI-resistant Ph+ acute lymphoblastic leukemia (Ph+ ALL) cells was related to Bcl-6 activity and the pathogenesis of ALL was closely associated with the Bcl-6/Arf/p53 pathway signals [28]. We showed that Bcl-6 was over expressed in K562/G01 cells, which indicates Bcl-6 overexpression is closely linked to TKI resistance in CML.

*Bcl-6* plays an important part in the growth and differentiation of B cells, is associated with pathogenesis of DLBCL [29], and promotes CML stem cell survival by inhibiting apoptosis. It has been reported that *Bcl-6* can repress *p53*-dependent apoptosis because of DNA damage [30], and that *MicroRNA-187* can induce apoptosis of DLBCL cells by downregulating Bcl-6 expression [31]. Qiang et al. demonstrated the oncogenetic properties of Bcl-6 in breast cancer, and concluded that Bcl-6 could be a target in the treatment of breast cancer [32]. In this study, we showed that low Bcl-6 expression inhibited growth and apoptosis of IM-resistant K562 cells, and more importantly, mediated high expression of p53, which induced apoptosis of tumor cells.

HHT is currently used as an effective treatment of hematological diseases, especially ALL [33]. We demonstrated that HHT inhibited growth, caused cell cycle arrest of IM-resistant K562 cells, and blocked the Bcl-6/p53 pathway.

Although we know Bcl-6 is associated with IM-resistant CML, additional studies are needed to describe causes. Our research showed that the effects of HHT on drug resistance involved inhibition of the Bcl-6/p53 pathway.

## MATERIALS AND METHODS

### Bone marrow samples and patients

At the Department of Hematology, Huai’an First People's Hospital, Nanjing Medical University, Huai’an, China, bone marrow (BM) samples were collected from patients between April 2015 and May 2016. From patients with newly diagnosed CML in the chronic phase (CML-CP, n = 20) and those in blast crisis (CML-BP, n = 10), we acquired the bone marrow samples. Healthy control samples were from 11 volunteers without CML. Mononuclear cells from the samples were isolated by Ficoll–Hypaque density gradient centrifugation, after that put in −80°C. The Ethics Committee of Nanjing Medical University approve the research. The IM was obtained from Selleck and the HHT from MedChem Express (MCE, Shanghai, China).

### Transfection and cell culture

The CML cell lines K562 and K562/G01 (Cell Repository, Chinese Academy of Science, Shanghai, China) was incubated at 37°C, with 95% air and 5% CO_2_ in RPMI 1640 (Gibco, USA) containing 10% fetal bovine serum (FBS, Gibco). The human CML cell line KU812 (Basic Medical Institute, Chinese Academy of Medical Sciences, Beijing, China) was incubated at 37°C, with 95% air and 5% CO_2_ in RPMI 1640 containing 10% FBS. The human Ph+ ALL cell line SUP-B15 (Cell Repository, Chinese Academy of Science, Shanghai, China)was incubated at 37°C, with 95% air and 5% CO_2_ in Iscove's Modified Dulbecco's Medium (IMDM, Gibco) containing 10% FBS. The human renal epithelial cell line 293T (Cell Repository, Chinese Academy of Science, Shanghai, China) was cultured at 37°C, with 95% air and 5% CO_2_ in DMEM (Gibco) containing 10% FBS. Ficoll density gradient centrifugation (TBD Science, Tianjin, China) isolate blast cells. The K562/G01 cells were transfected with *Bcl-6* siRNA using Lipofectamine 2000 (Invitrogen, USA) for 48 h. *Bcl-6* siRNA was synthesized by GenePharmagps in Shanghai. The sequences for the *Bcl-6* siRNA1 were:5′-GCAGUUUAGAGCCCAUAAATT-3′ (sense), 5′-UUUAUGGGCUCUAAACUGCTT-3′ (antisense). Those for *Bcl-6* siRNA2 were: 5′-GCAAUAUCUAUUCACCCAATT-3′ (sense), 5′-UUGGGUGAAUAGAUAUUGCTT-3′ (antisense).

**Table:** 

	siRNA sequences
*Bcl-6 siRNA1(sense)*	5′-GCAGUUUAGAGCCCAUAAATT-3′
*Bcl-6 siRNA1(antisense)*	5′-UUUAUGGGCUCUAAACUGCTT-3′
*Bcl-6 siRNA2(sense)*	5′-GCAAUAUCUAUUCACCCAATT-3′
*Bcl-6 siRNA2(antisense)*	5′-UUGGGUGAAUAGAUAUUGCTT-3′

### Real-time PCR and RNA extraction

The total RNA in cells and human BM samples was extracted using TRIzol (Invitrogen, USA). The mRNA isolation kit extracted total RNA following the reagent supplies manual. For the mRNA quantitative analysis, reverse-transcribed the RNA using the NCode™ SYBR Green kit (Invitrogen, USA). All qPCR was carried out with the Bio-Rad CFX96. qPCR arrays were obtained from SABiosciences and used according to the reagent supplies manual. The comparative threshold cycle (Ct) method was used to analyze every sample. The levels of expression of *Bcl-6* and *p53* were normalized to that of *GAPDH*. The PCR primer sequences were for *Bcl-6*: 5′-TCCAGTCCCCACTCACTCAC-3′ (forward) and 5′-TTGCTCAAAACCAAATGAGCACT-3′ (reverse); for *p53*: 5′-TTATGGCGGGAGGTAGACTG-3′ (forward) and 5′-GTTCCGAGAGCTGAATGAGG-3′ (reverse); for *GAPDH*: 5′-ACCAGCCTCAAGATCATCAGC-3′ (forward) and 5′-TGCTAAGCAGTTGGTGGTGC-3′ (reverse). The expression change calculate relative to the control (2-ΔΔCt).

**Table:** 

	primer sequences
*Bcl-6(forward)*	5′-TCCAGTCCCCACTCACTCAC-3′
*Bcl-6(reverse)*	5′-TTGCTCAAAACCAAATGAGCACT-3′
*p53(forward)*	5′-TTATGGCGGGAGGTAGACTG-3′
*p53 (reverse)*	5′-GTTCCGAGAGCTGAATGAGG-3′
*GAPDH (forward)*	5′-ACCAGCCTCAAGATCATCAGC-3′
*GAPDH (reverse)*	5′-TGCTAAGCAGTTGGTGGTGC-3′

### Western blot analysis

RIPA lysis buffer was used to isolate proteins (Beyotime, Shanghai, China), and the Bradford method determine protein concentrations. Proteins (20 μg) were divided by SDS-polyacrylamide gel electrophoresis (SDS-PAGE, Beyotime) and transferred onto PVDF membranes (Millipore, USA). After 2h blocking in 5% bovine serum albumin (BSA, Vicmed, Xuzhou, China), the membranes were overnight incubated in primary antibody, diluted at 4°C in 5% BSA.

The primary antibodies were:anti-Bcl-6 (1:1000, Abcam, USA), anti-caspase3 (1:1000, Proteintech, USA), and anti-GAPDH (1:5000, Bioworld), anti-p53 (1:1000, Proteintech), anti-caspase9 (1:1000, Bioworld, Shanghai, China). Washing, then incubating the membranes with Horseradish Peroxidase (HRP) -conjugated rat anti- rabbit immunoglobulin (1/10000) (1:5000, Bioworld) for 1 h, diluted in 5% BSA. After washing, the secondary antibody was visualized by an enhanced chemiluminescence kit (Vicmed).

### Flow cytometry for apoptotic analysis

PE *Annexin V* & PI staining (BD Biosciences, USA) evaluate apoptosis. Cells (10^6^) use PBS (Gibco) to wash twice and in binding buffer resuspend. Working solution was put into the cell suspension, cells were incubated 15 minutes at room temperature by the reagent supplies manual. Flow cytometry determine the apoptotic cells percentage.

### Flow cytometry for cell cycle analysis

Cells were assessed by PI staining (BD Biosciences). Cells (10^6^) use PBS (Gibco) washed twice and were resuspended in PBS (Gibco) once. 70% prechilled ethanol fix cells overnight at 4°C, washed with prechilled PBS, and handled with 300 μl staining solution containing 50 μg/ml PI and 50μg/ml RNase A in dark for 30 minutes in turn. Then, DNA content was measured via FACSCalibur flow cytometry (BD Biosciences). The data were analyzed with the ModFit DNA analysis program.

### CCK-8 assay

CCK8 (Dojindo, Tokyo, Japan) analyze the tolerance IM concentrations in two cells. The two cells were plated into 96-well plates at 1×10^4^ cells/well in 100 μl agent. For pre-incubation 24h, IM were added at various doses. In every test cells treated with the same concentrations of dimethyl sulfoxide (DMSO) were contained. Controls are undisposed cells. Inhibition rate was calculated as [1 – (OD treated – OD blank)/(OD control – OD blank)] ×100%.

### Statistical analysis

SPSS 20.0 statistical software analyzed the statistical. The mean of three independent tests are represented by values and values are demonstrated as mean ±SD. An independent-samples t test was analyzed the compare of the proliferation and apoptosis of cells following IM or HHT intervention. A *P* value <0.05 was the accepted level for significance.
